# Role of GP82 in the Selective Binding to Gastric Mucin during Oral Infection with *Trypanosoma cruzi*

**DOI:** 10.1371/journal.pntd.0000613

**Published:** 2010-03-02

**Authors:** Daniela I. Staquicini, Rafael M. Martins, Silene Macedo, Gisela R. S. Sasso, Vanessa D. Atayde, Maria A. Juliano, Nobuko Yoshida

**Affiliations:** 1 Departamento de Microbiologia, Imunologia e Parasitologia, Universidade Federal de São Paulo, São Paulo, Brasil; 2 Departamento de Morfologia, Universidade Federal de São Paulo, São Paulo, Brasil; 3 Department of Internal Medicine and Cell Biology, Yale University Medical School, New Haven, Connecticut, United States of America; 4 Departamento de Biofísica, Universidade Federal de São Paulo, São Paulo, Brasil; New York University School of Medicine, United States of America

## Abstract

Oral infection by *Trypanosoma cruzi* has been the primary cause of recent outbreaks of acute Chagas' diseases. This route of infection may involve selective binding of the metacyclic trypomastigote surface molecule gp82 to gastric mucin as a first step towards invasion of the gastric mucosal epithelium and subsequent systemic infection. Here we addressed that question by performing in vitro and in vivo experiments. A recombinant protein containing the complete gp82 sequence (J18), a construct lacking the gp82 central domain (J18*), and 20-mer synthetic peptides based on the gp82 central domain, were used for gastric mucin binding and HeLa cell invasion assays, or for in vivo experiments. Metacyclic trypomastigotes and J18 bound to gastric mucin whereas J18* failed to bind. Parasite or J18 binding to submaxillary mucin was negligible. HeLa cell invasion by metacyclic forms was not affected by gastric mucin but was inhibited in the presence of submaxillary mucin. Of peptides tested for inhibition of J18 binding to gastric mucin, the inhibitory peptide p7 markedly reduced parasite invasion of HeLa cells in the presence of gastric mucin. Peptide p7*, with the same composition as p7 but with a scrambled sequence, had no effect. Mice fed with peptide p7 before oral infection with metacyclic forms developed lower parasitemias than mice fed with peptide p7*. Our results indicate that selective binding of gp82 to gastric mucin may direct *T. cruzi* metacyclic trypomastigotes to stomach mucosal epithelium in oral infection.

## Author Summary

Frequent outbreaks of acute Chagas' disease by food contamination with *T. cruzi*, characterized by high mortality, have been reported in recent years. In Brazil, oral infection is currently the most important mechanism of *T. cruzi* transmission. Studies on oral *T. cruzi* infection in mice have shown that insect-stage metacyclic trypomastigotes invade only the gastric mucosal epithelium and not other areas of mucosal epithelia prior to establishing systemic infection. Here we have shown that metacyclic trypomastigotes bind selectively to gastric mucin, a property also displayed by gp82, a metacyclic stage-specific surface protein implicated in cell adhesion/invasion process. It is also shown that the gastric mucin-binding property of gp82 resides in the central domain of the molecule and that the synthetic peptide p7, based on a gastric mucin-binding sequence of gp82, markedly reduces parasite invasion of cultured human epithelial cells in the presence of gastric mucin. These results, plus the finding that mice that received peptide p7 before oral infection with metacyclic trypomastigotes had fewer parasites replicating in the gastric mucosa and developed lower parasitemias than control mice, lead us to suggest that gp82-mediated interaction with gastric mucin may direct *T. cruzi* to stomach mucosal epithelium in oral infection.

## Introduction

Orally transmitted infection by the protozoan parasite *Trypanosoma cruzi* has been responsible for frequent outbreaks of acute cases of Chagas' disease in recent years [1],[2]. In Brazil, after the elimination of the domiciliary vector *Triatoma infestans* in many endemic areas, and the control of the blood bank transmission, *T. cruzi* infection by the oral route constitutes the most important transmission mechanism [2]. The occurrence of Chagas' disease through food contamination, involving triatomine insects other than *T*. *infestans*, has been reported in different geographical regions. The incidence is higher in the Brazilian Amazon [1], where more than half of acute cases of the disease reported in the last 40 years can be attributed to microepidemics of orally transmitted infection [2],[3].

Studies on oral *T. cruzi* infection in the mouse model have shown that the insect stage metacyclic trypomastigotes invade the gastric mucosal epithelium and, following intracellular replication as amastigotes, differentiate into trypomastigotes that are subsequently released into circulation [4],[5]. During oral infection, gastric mucosa is uniquely targeted for metacyclic trypomastigote entry in order to establish a systemic *T. cruzi* infection, with parasites being undetectable elsewhere within the mucosa of the oropharynx or esophagus [4]. There are several evidences that the metacyclic stage-specific surface glycoprotein gp82 plays a critical role in the establishment of *T. cruzi* infection by the oral route [6],[7]. Gp82 is a cell adhesion molecule that mediates metacyclic trypomastigote entry into cultured human epithelial cells, by triggering the signal transduction pathways leading to cytosolic Ca^2+^ mobilization in both cells [8], an event essential for parasite internalization [9],[10],[11]. In addition to cell invasion-promoting properties, gp82 has the ability to bind to gastric mucin [6]. Through gp82-mediated interaction with gastric mucin, a constituent of the luminal barrier that functions as a first line of defense against invading pathogens, the parasites may effectively be addressed to the target cells. Metacyclic forms of *T. cruzi* strains deficient in gp82 expression are poorly infective when administered orally into mice, although they efficiently invade host cells in vitro by engaging gp30, a Ca^2+^ signal-inducing surface molecule related to gp82 but devoid of gastric mucin-binding property [7]. Unlike gp82-expressing strains, the gp82-deficient strains have reduced capacity to enter cultured epithelial cells in the presence of gastric mucin [7]. This reinforces gp82 binding to gastric mucin as an important requirement for parasites reaching the underlying target cells. Selective binding of gp82 to gastric mucin could explain why parasite invasion is not found anywhere within the oropharynx or esophagus [4]. *Shigella dysenteriae*, for instance, whose pathogenic potential correlates with its capacity to invade and multiply within cells of the colonic epithelium, adheres preferentially to colonic mucin [12]. Although purified gp82, either in its native form or as a recombinant protein, binds to gastric mucin, it remains to be demonstrated that *T. cruzi* metacyclic forms bind selectively to gastric mucin in gp82-dependent manner. Here we aimed at addressing that question, at identifying the gp82 sequences involved in gastric mucin-binding, and at investigating the effect of gp82-based synthetic peptides on metacyclic trypomastigote infection in vitro and on oral infection in mice.

## Methods

### Parasite and host cell invasion assay

*T. cruzi* strain CL [13] was used throughout. Parasites were maintained cyclically in mice and in liver infusion tryptose medium. Metacyclic trypomastigotes, generated in Grace's medium, were purified by passage through DEAE-cellulose column, as described [14]. HeLa cells, the human carcinoma-derived epithelial cells, were grown at 37°C in Dulbecco's Minimum Essential Medium, supplemented with 10% fetal calf serum, streptomycin (100 µg/ml) and penicillin (100 U/ml) in a humidified 5% CO_2_ atmosphere. Cell invasion assays were carried out as detailed elsewhere [15], by seeding the parasites onto each well of 24-well plates containing 13-mm diameter round glass coverslips coated with 1.5×10^5^ HeLa cells. After 1 h incubation with parasites, at multiplicity of infection 10∶1, the duplicate or triplicate coverslips were fixed in Bouin solution, stained with Giemsa, and sequentially dehydrated in acetone, a graded series of acetone∶xylol (9∶1, 7∶3, 3∶7) and xylol. Cell invasion assays in the presence of gastric mucin were performed with mucin suspended in culture medium.

### Production and purification of recombinant proteins J18, J18b, J18* and C03

The recombinant protein J18, containing the full-length *T. cruzi* gp82 (GenBank accession number L14824) in frame with gluthatione S-transferase (GST), was produced in *E. coli* DH5-α by transforming the bacteria with a pGEX-3 construct comprising the *gp82* gene [16]. All steps for induction of the recombinant protein J18 and its purification are detailed elsewhere [17]. J18b, a construct containing the carboxy-terminal half of gp82, and J18*, with deletion of 65 amino acids (residues 257 to 321), were prepared as previously described [18],[19] and purified in the same manner as J18. In addition, from a cloned full-length gp82 cDNA named C03 (GenBank accession number EF445668) we generated the recombinant protein C03 containing histidine tail, as previously described [17]. The amount of purified protein was quantified by reaction with Coomassie Plus (Pierce) in 96 well plates, and reading at 620 nm. To certify that the desired protein was obtained, the purified samples were analyzed in SDS-PAGE gel stained with Coomassie Blue, and by immunoblotting using anti-GST antibodies.

### Binding of recombinant proteins based on metacyclic trypomastigote gp82 to mammalian mucin

Microtiter plates (96 wells) were coated with mucin from porcine stomach (Type III, Sigma) or from bovine submaxillary glands (Type I-S, Sigma) in PBS (10 µg/well). After blocking with PBS containing 2 mg/ml bovine serum albumin (PBS/BSA) for 1 h at 37°C, the plates were sequentially incubated at 37°C for 1 h with the recombinant protein J18, J18* or C03, with polyclonal monospecific antibody directed to J18, C03 or GST, and with peroxidase-conjugated anti-mouse IgG, all diluted in PBS/BSA. The final reaction was revealed by *o*-phenilenediamine and the absorbance at 492 nm read in a Multiscan MS ELISA reader. To check the effective coating with mucin, the mucin-coated microtiter plates (10 µg/well) were blocked with PBS/BSA and incubated at 37°C for 1 h with anti-gastric mucin or anti-submaxillary mucin antisera diluted 1∶100 in PBS/BSA. Reaction proceeded with peroxidase-conjugated anti-mouse IgG, as described above. For coating with gastric mucin preparation at pH 2.5 citrate buffer was used.

### Competitive gastric mucin-binding between the recombinant protein J18 and synthetic peptides

Wells of microtiter plates were coated with gastric mucin (10 µg/well). After blocking with PBS/BSA, the plates were incubated for 1 h at 37°C with J18 (10 µg/ml) in absence or in the presence of individual synthetic peptides (200 µg/ml) corresponding to the gp82 sequence spanning residues 224–333, synthesized as described [19]. Following incubation with anti-GST antibodies and peroxidase-conjugated anti-mouse IgG, the reaction was revealed by *o*-phenilenediamine.

### *T. cruzi* metacyclic trypomastigote binding to mammalian mucin and inhibition by J18

Twenty four- well plates, containing 13 mm round glass coverslips, were incubated overnight at 37°C with 100 µl of gastric mucin or submaxillary mucin, at 400 µg/ml in PBS. After washings in PBS, a metacyclic trypomastigote suspension in cell culture medium (5×10^7^/ml) was added to each well and incubation proceeded for 1 h at 37°C. Following washes with PBS, the coverslips were fixed in methanol and Giemsa-stained for microscopic visualization of parasites. ELISA assay for metacyclic trypomastigote binding to mammalian mucin was performed as follows: 96-well microtiter plates coated with gastric mucin or submaxillary mucin, at varying concentrations in PBS, were washed in PBS and then 50 µl of parasite suspension in cell culture medium (5×10^7^/ml) were added. Following 1 h incubation at 37°C and washings in PBS, the parasites were fixed with 3.5% formaldehyde for 20 min at room temperature. After washings in PBS, the parasites were sequentially incubated with a monoclonal antibody to *T. cruzi* surface glycoprotein gp35/50, and peroxidase-conjugated anti-mouse IgG, all of them diluted in PBS/BSA. The final reaction was revealed by *o*-phenilenediamine. The same protocol was used for inhibition of parasite binding to gastric mucin, in which microtiter plates coated with gastric mucin (10 µg/well) were incubated with metacyclic forms in absence or in the presence of varying concentrations of the recombinant protein J18 or GST.

### Parasite migration assay through mucin layer

Polycarbonate transwell filters (3 µm pores, 6.5 mm diameter, Costar) were coated with 50 µl of a preparation containing 10 mg/ml gastric mucin or submaxillary mucin in water. *T. cruzi* metacyclic trypomastigotes, in 600 µl PBS were added to the bottom of 24-well plates (7.5×10^7^ parasites/well) and incubated for 1 h at 37°C. Thereafter, the mucin-coated transwell filters were placed onto parasite-containing wells, and 100 µl PBS were added to the filter chamber. At different time points of incubation at 37°C, 10 µl were collected from the filter chamber for determination of parasite number and the volume in this chamber was corrected by adding 10 µl PBS.

### Oral infection

Four to five week-old female Balb/c mice, bred in the animal facility at Universidade Federal de São Paulo, were used. All procedures and experiments conformed with the regulation of the institutional Ethical Committee for animal experimentation, and the study was approved by the Committee. Mice were infected with *T. cruzi* metacyclic forms by oral route (4×10^5^ parasites per mouse), using a plastic tube adapted to a 1 ml syringe. Starting on day 10 post-inoculation, parasitemia was monitored twice a week by examining 5 µl blood samples collected from the tail, at the phase contrast microscope. For detection of parasites in the gastric mucosal epithelium, the stomach of mice inoculated orally with metacyclic forms was collected, fixed with 10% neutral formaldehyde for 24 h. After processing by gradual dehydration in a graded series of ethanol solution, followed by xylene immersion and embedding in parafilm, serial 5 µm tissue sections were cut and stained with hematoxylin and eosin.

### Antibodies

Six to eight week-old Balb/c mice were used for immunization with J18, C03, GST, gastric mucin or submaxillary mucin to generate specific antibodies. Mice received the first dose of antigen (10 µg/mouse) adsorbed in Al(OH)_3_ (0.5 mg/mouse) and after two weeks received three additional doses of the antigen plus the same adjuvant at one week interval. Ten days after the last immunizing dose, the mice were bled by heart puncture and the serum collected.

### Statistical analysis

To determine significance of data by Student's *t* test, the program GraphPad InStat was used.

## Results

### *T. cruzi* metacyclic trypomastigotes selectively bind to gastric mucin, traverse the gastric mucin layer and invade the underlying target cells

**Figure 1:**
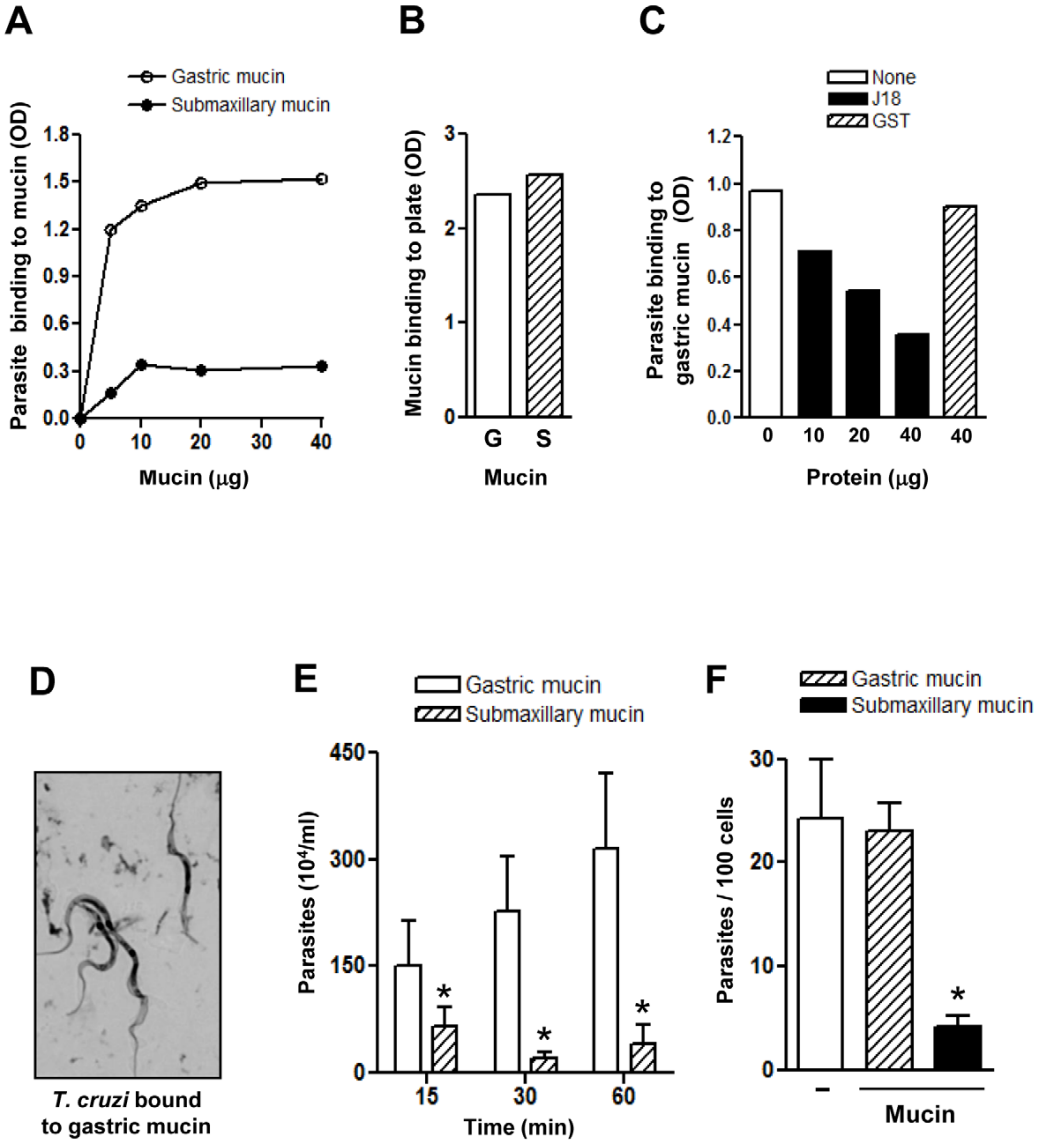
Selective binding of *T. cruzi* metacyclic trypomastigotes to gastric mucin. A) Microtiter plates coated with varying amounts of gastric or submaxillary mucin were incubated with metacyclic forms for 1 h and processed for detection of bound parasites by ELISA. Values are the means of triplicates of one representative assay out of three. Variation between triplicates was <5%. B) Microtiter plates coated with gastric (G) or submaxillary (S) mucin (10 µg/well) were processed for ELISA using antibodies specific for gastric or submaxillary mucin at 1∶100 dilution. Values are the means of triplicates (variation <5%). C) Metacyclic forms were added to microtiter plates coated with gastric mucin (10 µg/well) and incubated in absence or in the presence of the indicated amounts of the recombinant protein J18 or GST and processed for ELISA. Values are the means of triplicates. (variation <10%). D) Metacyclic trypomastigotes were added to gastric mucin-coated coverslips and, following the procedure described in the methods section, the Giemsa-stained parasites were visualized under the microscope. E) Transwell filters coated with gastric or submaxillary mucin were placed onto wells containing metacyclic forms. At different time points, samples from the filter chamber were collected and the number of parasites counted. Values represent the means ± standard deviation of three independent experiments. The difference in parasite translocation through gastric and submaxillary mucin layer was significant (*), with P<0.05. F) Gastric or submaxillary mucin was added to HeLa cells before addition of metacyclic trypomastigotes. After 1 h at 37°C, the cells were fixed and Giemsa-stained. The number of internalized parasites was counted in a total of 250 cells. Values correspond to means ± SD of 4 independent experiments. There was a significant difference between invasion in absence and in the presence of submaxillary mucin (*), P<0.01.

To address the question why, upon oral inoculation into mice, *T. cruzi* metacyclic forms invade the gastric mucosa but not the mucosa of the oropharynx [4], we examined the possibility that metacyclic forms bind to gastric mucin but not to submaxillary mucin. Assays were performed by incubating microtiter plates coated with varying amounts of gastric or submaxillary mucin with parasites at 37°C for 1 h, followed by fixation. Reaction with a monoclonal antibody to *T. cruzi* surface glycoprotein gp35/50 revealed that metacyclic trypomastigotes bound to gastric mucin in a dose-dependent manner whereas binding to submaxillary mucin was negligible at all concentrations tested (Fig. 1A). Effective coating with mucins was ascertained by reaction with antibodies specific for gastric or submaxillary mucin (Fig. 1B). To determine whether metacyclic surface molecule gp82 was implicated in binding to gastric mucin, assays were also performed in the presence of a recombinant protein containing the full length gp82 sequence fused to GST (J18). Metacyclic trypomastigote binding to gastric mucin was inhibited in a dose-dependent manner by J18 but not by GST (Fig. 1C), consistent with a role for gp82. Parasites bound to gastric mucin were visualized by microscopic examination in parallel assays using coverslips coated with gastric mucin (Fig. 1D), as described in the methods section. In addition, the ability of metacyclic forms to traverse a mucin layer was examined in microtiter plates with transwell filters coated with gastric or submaxillary mucin, by counting the number of parasites that translocated through the mucin layer at varying time points. As shown in Fig. 1E, the number of parasites that traversed the gastric mucin layer was significantly higher than the number of parasites that traversed the submaxillary mucin layer. Next, we determined the ability of metacyclic forms to enter host cells in the presence of gastric or submaxillary mucin, in an attempt to mimic the in vivo situation in which the parasites interact with and traverse the mucous layer before reaching the underlying epithelial cells. Mucin, at 2 mg/ml in culture medium, was added to HeLa cells 15 min before addition of parasites. Following 1 h incubations with parasites, the cells were fixed, stained, and the number of intracellular parasites was counted. The rate of metacyclic trypomastigote internalization was comparable in the absence or in the presence of gastric mucin, but was drastically reduced in the presence of submaxillary mucin (Fig. 1F). Even at 20 mg/ml, gastric mucin did not exhibit any inhibitory effect on parasite entry into HeLa cells.

### Binding of J18 to gastric mucin is specific and requires the central domain of the molecule

**Figure 2:**
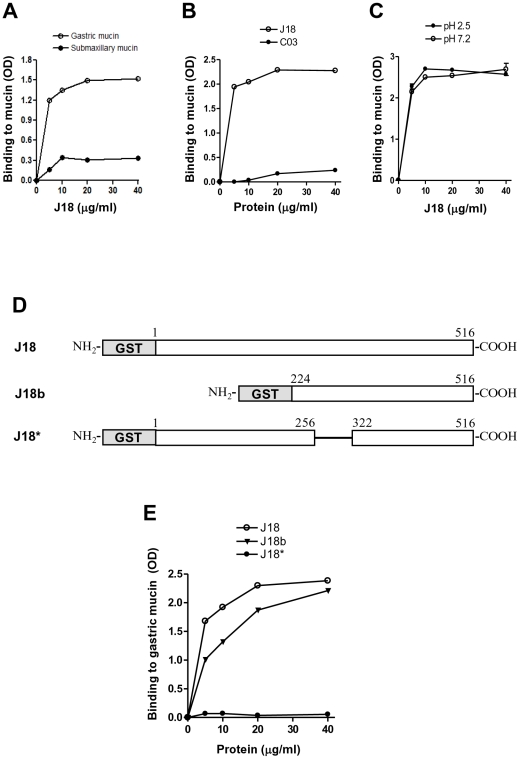
Specific binding of the recombinant protein J18 to gastric mucin through its central domain. A) The recombinant protein J18, containing the full length *T. cruzi* gp82 peptide sequence, was added to microtiter plates coated with gastric or submaxillary mucin at the indicated concentrations. Values are the means of triplicates of one representative assay out of three. Variation between triplicates was <5%. (B) C03, a recombinant protein of *T. cruzi* gp82 family with 59.1% identity with J18, was used for gastric mucin-binding assay. C) Gastric mucin preparations at pH 2.5 and pH 7.2 were used to coat microtiter plates and then binding of J18 was performed. In (B) and (C), the values are the means of triplicates (variation <5%). D) Schematic representation of recombinant proteins based on gp82 molecule. Shown are the GST-fused constructs containing the full-length gp82 sequence (J18) or lacking either the amino-terminal portion (J18b) or the central domain spanning residues 257–321 (J18*). E) Binding of J18, J18b and J18* to gastric mucin was compared. Values are the means of triplicates (variation <10%).

To ascertain that the metacyclic trypomastigote surface molecule gp82 bound preferentially to gastric mucin when compared to submaxillary mucin, ELISA assays were carried out using J18, the recombinant protein containing the full-length gp82 sequence, previously shown to bind to gastric mucin in the same manner as the native molecule [6]. J18 bound to gastric mucin in a dose-dependent and saturable manner whereas binding to submaxillary mucin was minimal (Fig. 2A). As gp82 is encoded by a multigene family [20], we asked whether another member of the family also possessed gastric mucin-binding property. When compared to J18, the gastric mucin-binding ability of the recombinant protein C03, which shares 59.1% sequence identity with J18 [17] was negligible (Fig. 2B). Other metacyclic surface molecules, such as gp90 and gp35/50, were devoid of gastric mucin binding capacity (data not shown). A question that was also examined concerned the effect of pH on J18 binding to gastric mucin. It has recently been reported that pH affects the association behavior of pig gastric mucin in aqueous solutions, with large interchain aggregates being detectable at pH 2 by dynamic light scattering [21]. Gastric mucin preparations at pH 7.2 and at pH 2.5, which is close to the pH of the gastric milieu, were used to coat microtiter plates for J18 binding assays. As shown in Fig. 2C, J18 bound efficiently to gastric mucin regardless of the pH. To determine the gastric mucin-binding domain of the gp82 molecule, two other recombinant proteins were used: J18b, containing the carboxy-terminal half of gp82 and J18*, lacking 65 residues (amino acids 257 to 321) corresponding to the gp82 domain required for host cell adhesion (Fig. 2D). J18* did not bind to gastric mucin, in contrast to J18 and J18b (Fig. 2E).

### Gp82 sequences spanning amino acid residues 284–303 (p7) and 314–333 (p10) constitute the main gastric mucin-binding sites

**Figure 3:**
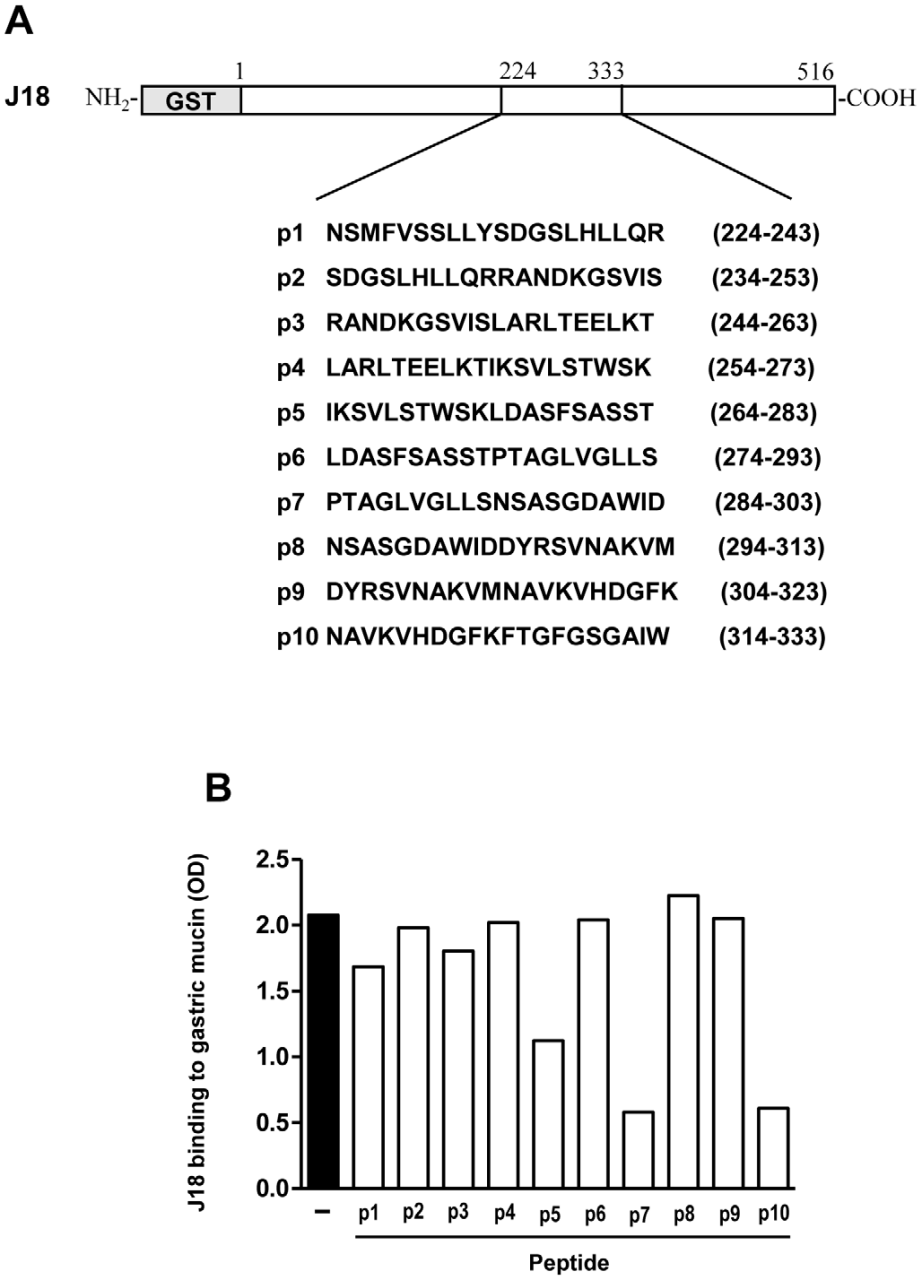
Determination of *T. cruzi* gp82 sequences implicated in binding to gastric mucin. A) Amino acid composition of peptides corresponding to gp82 central domain. Shown are the 20-mer peptides with an overlap of 10 residues. B) Effect of peptides shown in (A) on J18 binding to gastric mucin. Values are the means of triplicates of representative assays (variation between triplicates <10%).

To determine more precisely the gp82 sequence involved in binding to gastric mucin, we performed assays of competition between the recombinant protein J18 and synthetic peptides spanning the gp82 central domain. Gastric mucin-coated microtiter plates were incubated with J18 (10 µg/ml) in absence or in the presence of each of the 20-mer peptides with 10 overlapping residues, spanning amino acids 224–333 (Fig. 3A), and the reaction proceeded as in conventional binding assays. Peptides p7 and p10 inhibited J18 binding to gastric mucin by >70%, inhibition by p5 was on the order of ∼46% and by p1<20%, whereas other peptides showed no effect (Fig. 3B).

### Host cell invasion by metacyclic trypomastigotes is inhibited by peptide p7 in the presence of gastric mucin

**Figure 4:**
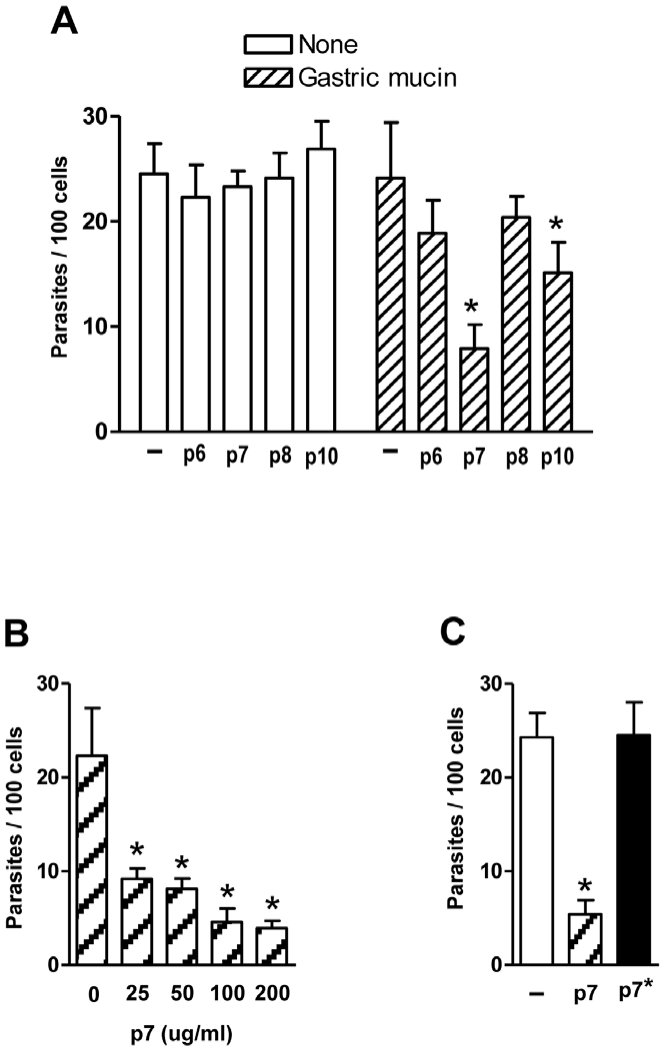
Peptide p7 inhibits host cell invasion by metacyclic trypomastigotes in the presence of gastric mucin. A) Parasites were incubated with HeLa cells in absence or in the presence of gastric mucin, plus the indicated peptides at 200 µg/ml. After 1 h incubation, the cells were fixed and stained with Giemsa for quantification of internalized parasites. Values are the means ± SD of 4 independent experiments. The inhibitory effect of peptide was significant (*) for p7, P = 0.005, and for p10, P<0.05. B). Metacyclic trypomastigotes were incubated with HeLa cells in the presence of gastric mucin, plus peptide p7 at the indicated concentrations and the reaction proceeded as above. Values are the means ± SD of 3 independent experiments. The difference in invasion rate in absence and in the presence of p7 was significant (*) at all concentrations, P<0.05. C) HeLa cells were incubated with metacyclic forms in the presence of gastric mucin, plus peptide p7 or p7* which has the same composition of p7 but a scrambled sequence. Values are the means ± SD of 4 independent assays performed in duplicate or triplicates.

We tested whether the synthetic peptides p7 and p10, which displayed the highest inhibitory effect on J18 binding to gastric mucin (Fig. 3B) could interfere with target cell invasion by metacyclic forms. In absence of gastric mucin, neither of these peptides affected the parasite internalization, but in the presence of gastric mucin peptide p7, and to a lesser degree p10, had a significant inhibitory activity while peptides p6 and p8 used as controls had no effect (Fig. 4A). The inhibitory effect of p7 was dose-dependent (Fig. 4B). To ascertain the sequence-specificity of that effect, a peptide with the same amino acid composition of p7 but with a scrambled sequence (LADLAGWLSPSDVGGAINST), designated p7*, was also tested in cell invasion assays. As shown in Fig. 4C, peptide p7* was devoid of inhibitory activity.

### Peptide p7 affects the course of oral *T. cruzi* infection in mice

**Figure 5:**
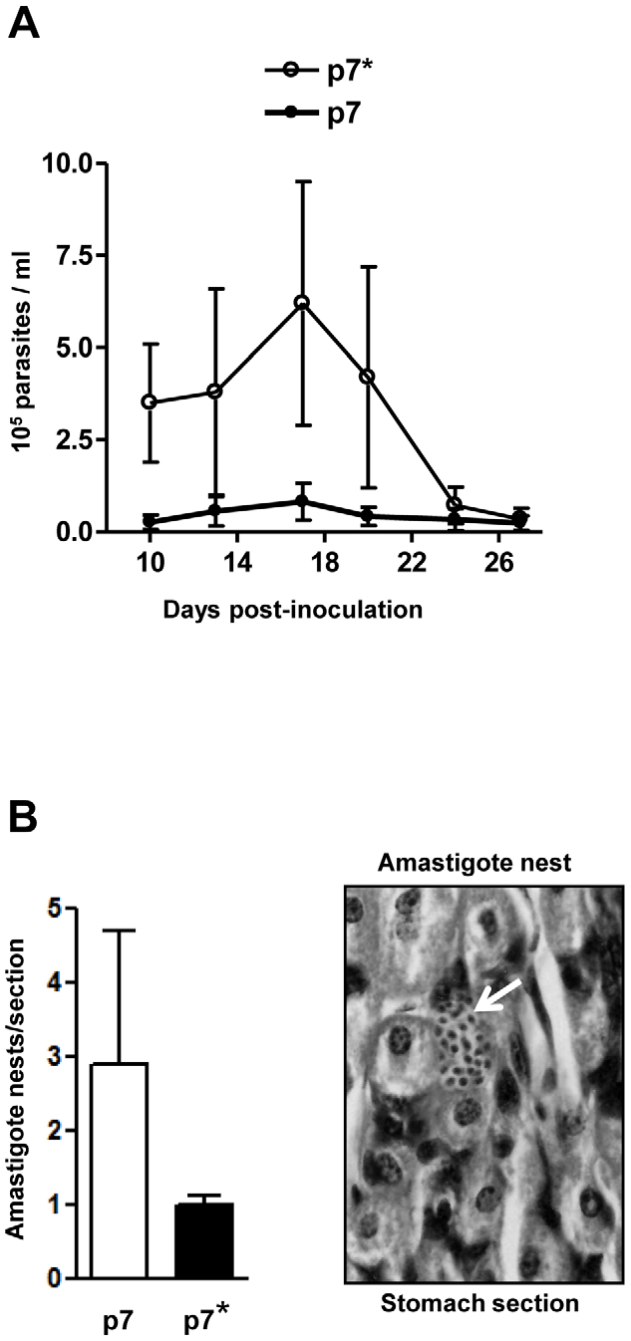
Inhibitory effect of peptide p7 on oral *T. cruzi* infection. A) Balb/c mice were divided in control (n = 5) and experimental (n = 5) groups, peptide p7* were given orally to control mice and peptide p7 to experimental animals 15 min before infection with metacyclic trypomastigotes by the oral route and the number of circulating parasites counted. Variations in the parasitemia levels between mice are indicated. B) Histological sections of the mouse stomach, collected 4 days after infection, were stained by hematoxylin and eosin and the number of amastigote nests (white arrow) was counted in 7 equivalent tissue sections. The representative results are shown, with bars corresponding to the variation in the number of parasites nests between sections.

To examine whether peptide p7 affected the in vivo metacyclic trypomastigote infectivity, Balb/c mice were administered with peptide p7 or p7* (20 µg/mouse) 15 min before oral infection with metacyclic forms (4×10^5^ parasites/mouse), and the parasitemia levels were monitored. Mice that received peptide p7 developed significantly lower parasitemias than those that received the control peptide p7* (Fig. 5A). To determine whether the difference in the parasitemia levels between the two groups resulted from differential invasion of gastric mucosal epithelium, the stomach of some mice was collected 4 days post-inoculation and processed for histological preparations. As shown in Fig. 5B, the number of parasites replicating in the gastric mucosa, visualized as amastigote nests in the stomach sections, was significantly lower in mice that received peptide p7 as compared to control mice that were given peptide p7* before parasite inoculation.

## Discussion

We had previously suggested that upon oral *T. cruzi* infection the metacyclic trypomastigote-specific surface molecule gp82 could play a key role in directing the parasites to the gastric mucosa, by promoting adhesion to mucin molecules that are the main constituent of the mucous layer, which would be traversed driven by ATP [22]. The results herein described provide support to that hypothesis.

Metacyclic trypomastigotes bound to gastric mucin in vitro and were capable of efficiently traversing the gastric mucin layer to invade the underlying epithelial cells. By contrast, the binding to mucin from submaxillary glands was minimal and the parasite ability to translocate through the submaxillary mucin layer, as well as the infectivity towards host cells in the presence of submaxillary mucin, were reduced. These results, in addition to the observations that metacyclic forms do not invade cells of the oropharynx after oral inoculation into mice [4], indicate that binding of metacyclic forms to gastric mucin is an important determinant in addressing the parasites to gastric mucosal epithelial cells. Metacyclic trypomastigotes of *T. cruzi* strains that bind poorly to gastric mucin have poor capacity to invade epithelial cells in vitro in the presence of gastric mucin and in infecting mouse by the oral route [7]. Selective binding to mucin molecules, as a prerequisite for the establishment of infection by other pathogenic microorganisms of the gastrointestinal tract have been reported. *Helicobacter pylori*, which colonizes gastric mucosa, binds to human gastric mucin [23], *Shigella*, which invades and multiplies within cells of the colonic epithelium, binds specifically to human colonic mucin but not to small intestine mucin [24].

Existing evidence suggests that binding of *T. cruzi* metacyclic trypomastigotes to gastric mucin is mediated by the surface molecule gp82. Binding of metacyclic forms to gastric mucin was inhibited by a recombinant protein based on gp82 in a dose-dependent manner. This finding, plus the previous observations that invasion of cultured epithelial cells by metacyclic forms of *T. cruzi* strains deficient in gp82 expression was reduced in the presence of gastric mucin [7] reinforce the idea that the gastric mucin-binding property of gp82 plays a key role in infection. Consistent with the in vitro findings, gp82-deficient metacyclic forms were poorly infective when administered orally into mice [7]. However, gp82 may not be the sole surface molecule that interacts with gastric mucin. We cannot rule out the possibility that other as yet unidentified metacyclic trypomastigote surface molecules bind to gastric mucin, albeit to a lesser extent.

The gastric mucin-binding domain of gp82 was found to be localized in the central domain of the molecule, and the peptide sequence PTAGLVGLLSNSASGDAWID was determined as being the most important for invasion of epithelial cells under the gastric mucin coat. In the presence of the synthetic peptide p7, based on that sequence, not only was the gastric mucin-binding of the recombinant gp82 inhibited but also the metacyclic trypomastigote invasion of epithelial cells coated with gastric mucin was reduced. Although displaying a similar inhibitory effect as p7 on gastric mucin-binding of the recombinant gp82, the peptide p10 (NAVKVHDGFKFTGFGSGAIW) affected parasite entry into target cells to a lesser degree than p7. One interesting possibility is that metacyclic trypomastigote binding to gastric mucin through the p7 defined region of gp82 facilitates host cell adhesion, but that adhesion is also dependent upon co-operation from other regions of gp82. The main cell adhesion site of gp82 is the sequence LARLTEELKTIKSVLSTWSK represented by peptide p4 [19], from which the p7 sequence is separated by 5 residues. Of note is that the isoelectric point of p4 is 9.71 and that of p7 is 3.23. The relevance, if any, of such a difference for target cell attachment is not known. In a previous study, a recombinant construct corresponding to the complete gp82 sequence, but without the two glutamic acid residues contained in p4 sequence, had reduced capacity to bind to HeLa cells [19]. Our speculative view is that metacyclic trypomastigotes bound to gastric mucin through the gp82 sequence p7 would have the p4 sequence-mediated interaction with host cells reinforced. If this is the case, the effective gp82-medianted parasite entry into target cells could be more than the result of independent contributions of diverse gp82 functional sites, p7 sequence acting in the early step of gastric mucin-binding and p4 sequence in the subsequent step of cell attachment.

It should be pointed out that the central domain of metacyclic trypomastigote gp82, where the p7 sequence resides, shares considerable sequence similarity with other glycoproteins of gp85 family and trans-sialidase, which are all members of the same superfamily. Analysis using BLASTP to search for sequences homologous to peptide p7 revealed proteins of gp85/trans-sialidase superfamily; therefore, it is possible that a motif similar to that represented by p7 may also be implicated in gastric mucin-binding and be responsible for the low proportion of cases in which cell invasion of metacyclic forms is not inhibited by p7 in the presence of gastric mucin (Fig. 4C). Another interesting possibility, unrelated to p7 sequence, is that metacyclic trypomastigote trans-sialidase binds to gastric mucin sialic residues, an interaction that is not inhibitable by peptide p7.

The importance of gp82-mediated binding to gastric mucin through the p7 sequence in the establishment of *T. cruzi* infection by the oral route was tested in the mouse model. Mice administered orally with peptide p7 prior to metacyclic trypomastigotes developed low parasitemia levels, in contrast to animals that received the control peptide p7*, with the same composition as p7 but with a scrambled sequence, which developed high parasitemias. The presence of much fewer nests of amastigotes replicating in the stomach epithelium in mice that were given p7, as compared to the number of amastigote nests in mice that received p7*, is an indication that fewer parasites invaded the gastric mucosal epithelium. What possibly occurs is that the presence of p7 interferes with gp82-binding to gastric mucin, precluding the parasite traversal of the mucus layer towards the target cells. Therefore, the gastric mucin-binding property of gp82 may be as critical for *T. cruzi* infection by the oral route as its cell-binding capacity.
