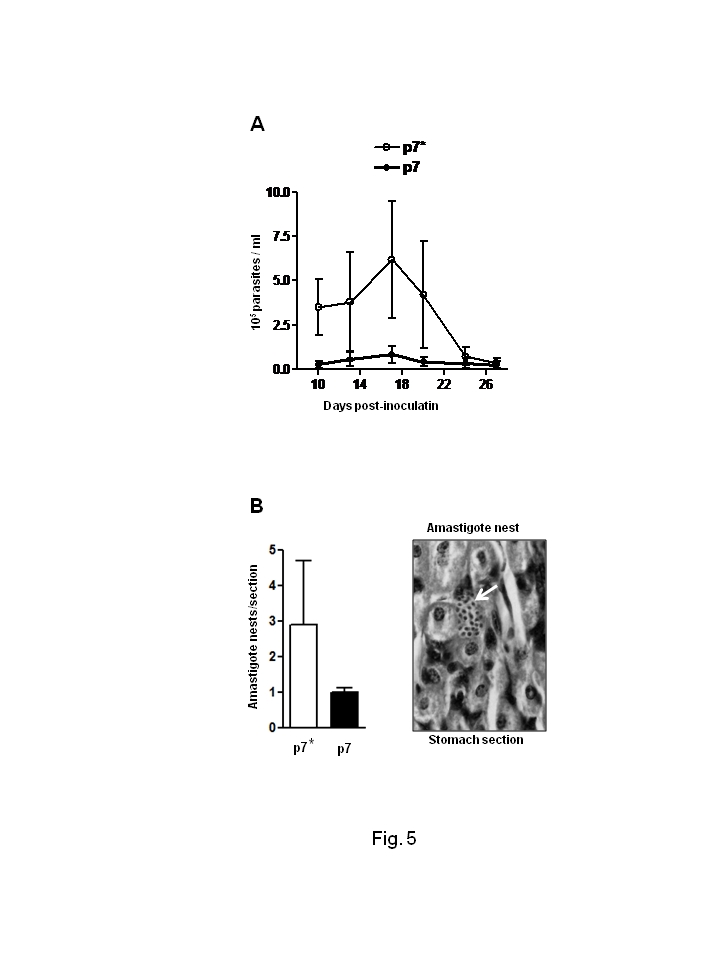# Correction: Role of GP82 in the Selective Binding to Gastric Mucin during Oral Infection with *Trypanosoma cruzi*

**DOI:** 10.1371/annotation/a81cf9ae-ac77-4f3b-a917-336d6616461d

**Published:** 2010-05-26

**Authors:** Daniela I. Staquicini, Rafael M. Martins, Silene Macedo, Gisela R. S. Sasso, Vanessa D. Atayde, Maria A. Juliano, Nobuko Yoshida

In Figure 5, part B, the labels for p7 and p7* were switched. Please see the corrected figure: 

**Figure:**